# Working-Condition-Indexed Generative Domain Generalization for Intelligent Fault Diagnosis Under Unseen Conditions

**DOI:** 10.3390/s26154693

**Published:** 2026-07-23

**Authors:** Duo Zhao, Fan Yang

**Affiliations:** Department of Automation, Tsinghua University, Beijing 100084, China; zhao_duo2022@163.com

**Keywords:** deep learning, domain generalization, fault diagnosis, adversarial learning

## Abstract

Domain generalization techniques have gained widespread application in cross-domain fault diagnosis in recent years. These methods enhance model generalization capabilities without accessing target domain data, thereby improving fault diagnosis performance under unknown operating conditions. However, most existing approaches overlook the fundamental relationships between operating conditions across different domains, resulting in directionless domain generalization. To address this limitation, we propose a working-condition-indexed generative domain generalization (WCIGDG) method for fault diagnosis in data-free conditions using given working-condition parameters. Within this framework, a domain index is employed to model relationships between operating conditions across domains. A domain index predictor is constructed to guide the data generation model, enabling the generated data to better adapt to the target operating condition, while adversarial training is introduced to enhance the effectiveness of data generation. Subsequently, the generated data is combined with source domain data to train the fault diagnosis model, strengthening its adaptability to the target operating condition. Experiments are conducted on two publicly available rotating machinery fault diagnosis datasets, namely the CWRU bearing dataset and the WT planetary gearbox dataset, using rotational speed as the domain index across 68 cross-speed domain generalization tasks. The results demonstrate the effectiveness of WCIGDG for both bearing and gear fault diagnosis. Compared with SDGN, the best-performing baseline among the compared methods, WCIGDG improves the average accuracy/F1-Score by 1.22/2.72 percentage points on CWRU and 1.33/1.93 percentage points on WT.

## 1. Introduction

Fault diagnosis of rotating machinery is essential for ensuring the reliability and safety of industrial equipment. Signal-processing methods, such as time-frequency analysis, envelope analysis, and physically interpretable feature extraction, have been widely used to identify fault-related spectral and modulation characteristics [1]. Digital-twin-based prognosis has also been explored by combining measured vibration data, signal processing, and crack-growth modeling to monitor fault severity and estimate the remaining time to maintenance [2]. These methods provide useful physical insight, but their performance often depends on expert-designed features, accurate prior models, or assumptions about operating conditions. To reduce the dependence on manual feature engineering and improve feature representation capability, deep-learning-based intelligent fault diagnosis has attracted increasing attention [3]. Such methods can automatically learn discriminative features from monitoring data, thereby improving the accuracy and efficiency of fault detection and localization.

Deep neural networks, however, demand substantial volumes of labeled training samples and require strict alignment between source and target domain data distributions to ensure model validity. In practical industrial application scenarios, equipment often operates under varying conditions, such as different loads and rotational speeds, potentially altering the data sample distribution. Moreover, due to the scarcity of labeled samples, especially fault samples, the target working conditions may encounter situations where data is unlabeled, biased, or even absent. Under these circumstances, models developed exclusively on source domain data tend to exhibit compromised generalizability when deployed in the target domain.

To address the issue of differing sample distributions caused by varying working conditions, domain adaptation techniques utilize labeled samples from the source domain and unlabeled samples from the target domain to derive a shared feature representation, consequently improving the model’s adaptability. For example, Ganin et al. [4] first proposed adversarial training based on a feature extractor and domain discriminator to obtain common features. Building on this, Chen et al. [5] integrated metric learning with adversarial learning to effectively address cross-machine fault diagnosis for ball screws in selective compliance assembly robot arms. To further tackle the challenges posed by data scarcity, Sun et al. [6] proposed a pseudo-label guided dual classifier domain adversarial network for unsupervised cross-domain fault diagnosis, which improves performance under small sample conditions by refining class boundaries.

Nevertheless, domain adaptation methods necessitate access to unlabeled target domain samples during model adaptation, thereby restricting practical deployment in industrial applications. In contrast, domain generalization methods are developed to acquire a model from one or multiple separate but related domains, such that the model exhibits robust generalization to unseen testing domains. Among many domain generalization methods, data manipulation and representation learning [7] are related to the method proposed in this paper. Data manipulation methods generate diverse samples through data augmentation and data generation to aid in generalization; for instance, Rahman et al. [8] used ComboGAN to generate new samples and employed an inter-domain distance metric function to reduce the distance between real and generated samples to learn common features. Zhou et al. [9] developed a strategy for generating multiple dissimilar domains to improve the model’s generalization capabilities. Within intelligent fault diagnosis systems, Fan et al. [10] utilized data augmentation methods across both class and domain spaces, applying adversarial learning mechanisms to improve cross-domain diagnostic robustness. Sun et al. [11] developed an autoregressive data generation method utilizing wavelet packet transform and cascaded stochastic quantization, effectively expanding unbalanced fault datasets to enhance diagnostic model training. Representation learning methods share similarities with domain adaptation techniques in that they strive to mitigate feature distribution divergences among multiple source domains in a specified latent space, thus improving cross-domain generalization performance for unseen operational environments. For example, Xie et al. [12] proposed an invariant feature purification method for domain generalization in rolling bearing fault diagnosis, and Song et al. [13] used a combination of covariance loss and graph convolutional networks to achieve feature enhancement for intelligent fault diagnosis. Recent advancements have also introduced dynamic network structures to better adapt to individual sample differences. Jiang et al. [14] proposed a semi-supervised dynamic generalization network (SDGN) featuring a dual feature enhancement strategy to improve the model’s adaptability to unseen working conditions.

Traditional domain generalization methods typically require access to labeled data from multiple source domains to enhance model generalizability. In fault diagnosis, related settings have been studied as semi-supervised domain generalization, where limited labeled source data are supplemented with unlabeled data from related working conditions. Recent research has focused on achieving semi-supervised domain generalization. For instance, Zhao and Shen [15] employed pseudo-labeling of unlabeled data alongside an entropy-based sample refinement mechanism to improve sample quality. Zeng et al. [16] utilized a joint learning strategy involving auxiliary and main branches to enhance model generalization capabilities. Li et al. [17] captured ambiguous boundary features by maximizing classifier discrepancies and leveraged adversarial training to obtain domain-invariant features, effectively adapting the model to diverse scenarios.

In many recent studies on domain adaptation, attention has been focused on the structural relationships between different domains to enhance the precision of domain adaptation. For instance, Wang et al. [18] used a continuous domain index to model the relationships between multiple source domains, Xu et al. [19] used graphs to model the relationships between different regions in multi-regional weather forecasting, Liu et al. [20] used categorical structures to model multiple source domains in image recognition domain adaptation, and Kumar et al. [21] progressively adapted domains in multiple gradually transformed source domains.

These studies indicate that the relationships among domains can provide useful guidance for cross-domain learning. However, most indexed-domain methods are developed for domain adaptation and therefore still require access to target-domain samples during training. This requirement limits their applicability to fault diagnosis scenarios in which the target working condition is known in advance, but vibration data from that condition are unavailable.

In domain generalization, including fault diagnosis applications, existing methods have primarily focused on improving model generalization by manipulating available data or learning invariant representations [22,23,24]. Although these methods can reduce distribution discrepancies, they often treat different working conditions as independent domains and make limited use of the physical relationships among them. In rotating machinery condition monitoring and fault diagnosis, measurable operating variables such as rotational speed and load have been shown to vary with operating conditions and to influence vibration or diagnostic features [25,26]. However, such physical working-condition information is rarely explicitly modeled in existing domain generalization methods.

To tackle this challenge, we define the problem of domain generalization of indexed domains and propose using a working-condition domain index to guide generative domain generalization methods. We construct the working-condition-indexed generative domain generalization (WCIGDG) method for fault diagnosis in data-free conditions based on given working-condition parameters. The contributions of this study are summarized as follows:

(1) To enhance the adaptability of generated data for specified operating conditions, we introduce the concept of a domain index in fault diagnosis problems. This index is employed to represent the gradual transition relationship of rotational speeds across different domains in rotating machinery. We propose constructing a domain index predictor to guide the data generation model.
(2) To strengthen the supervisory role of the domain index predictor and ensure the reliability of generated data, adversarial training is implemented between the generator and the domain index predictor. To enhance the stability of adversarial training, a weighted combination of multiple domain index predictors is employed.
(3) Based on this, we construct a fault diagnosis framework that can effectively improve the fault diagnosis performance for target domains with known working conditions but without data. The effectiveness and generalizability of the proposed framework are validated through 68 domain generalization tasks in two publicly available datasets.

## 2. Problem Formulation

We consider a setting in which a measurable working-condition parameter, such as rotational speed or load, exhibits an ordered or progressive variation across domains. When this parameter induces a systematic distribution shift, domains with more similar parameter values are expected to have more closely aligned data distributions. Such working-condition parameters can be represented by a domain index.

Let $U=U_{s}\cup U_{a}\cup U_{t}$ be a set of domain indices, where U is part of a metric space, and the domain index of any domain conforms to u∈U. $U_{s}$, $U_{a}$ and $U_{t}$ are the domain index sets for the source, auxiliary, and target domains, respectively. While the indices are drawn from a continuous space, each index set $U_{s}$, $U_{a}$, and $U_{t}$ comprises a discrete collection of elements.

The core hypothesis of our WCIGDG framework is that the conditional distribution P(X|u) evolves continuously with respect to the index u. In the context of machinery fault diagnosis, u represents physical operating parameters—such as rotational speed, torque, or temperature—that are associated with vibration signal characteristics and may systematically affect them. Consequently, the domain index serves as a distance metric: a smaller distance in the index space $\left\|u_{i}-u_{j}\right\|$ implies a smaller discrepancy (e.g., Wasserstein distance) between the feature distributions of domains i and j. This property allows the model to capture the functional relationship between the operating condition and the fault features, enabling generalization to unseen domains by interpolating or extrapolating along the learned manifold.

This assumption should be understood as an applicability condition rather than a universal property of all machinery signals. Any measurable working-condition parameter can be selected as the domain index when it produces a systematic distribution shift. In this study, rotational speed is adopted because the benchmark datasets vary primarily across speed conditions. If load, resonance, structural looseness, nonlinear contact, sensor position, or other coupled factors dominate the vibration response, a multidimensional or learned index may be required.

Similar to other domain generalization methods [27,28,29], we define the source domain and the target domain. The input and labels are denoted as x and y, respectively. The source domains are denoted as $D^{s}={\left\{D_{m}^{s}\right\}}_{m=1}^{M_{s}}$ where $D_{m}^{s}={\left\{(X_{s}^{m},Y_{s}^{m},u_{s}^{m})\right\}}_{m=1}^{M_{s}}$ denotes the mth source domain. The target domain is denoted as $D^{t}=\{X_{t},Y_{t},u_{t}\}$. Labeled data and the domain index are available in the source domain; the data and labels of the target domain are unseen, but its positional relationship within a series of domains can be represented by its domain index.

Unlike standard domain generalization approaches [30], we define a collection of auxiliary domains $D^{a}={\left\{D_{m}^{a}\right\}}_{m=1}^{M_{a}}$ where $D_{m}^{a}={\left\{(X_{a}^{m},Y_{a}^{m},u_{a}^{m})\right\}}_{m=1}^{M_{a}}$ denotes the mth auxiliary domain. Each auxiliary domain is equipped with domain indices and unlabeled data to assist in training the domain index predictor. Furthermore, it should be noted that in our WCIGDG framework, only a single source domain that is most similar to the target domain is selected for training the generator, while the remaining source domains serve as auxiliary domains.

Therefore, the objective of domain generalization of indexed domains is to utilize the data from a series of visible domains along with domain indices to train a robust model to accurately predict the labels $Y_{t}$ for the data-free target domain at a specified domain index $u_{t}$.

To avoid ambiguity, the main symbols used in WCIGDG are summarized in Table 1.

## 3. Proposed Method

**Figure 1:**
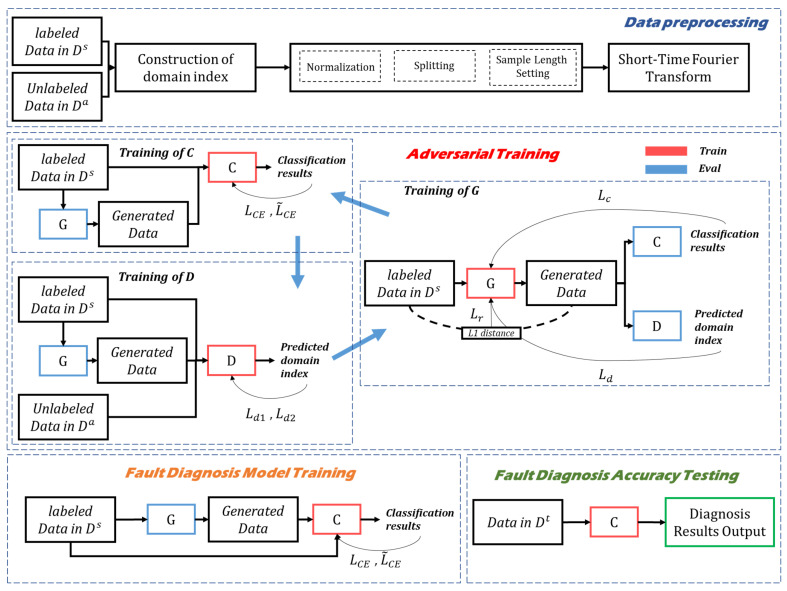
Structure of the proposed method. (Note: Dashed boxes group the main stages; black arrows and curves show data and loss paths, while blue arrows show the alternating optimization sequence among C, D, and G).

Figure 1 delineates the architecture of the proposed WCIGDG method, which comprises four distinct modules: data preprocessing, generator, domain index predictor, and fault diagnosis classifier.

We first analyze the operational condition distribution across all domains to determine the range of domain indices. Subsequently, based on the distance relationship of the domain indices, we select the domain with labeled data that is closest to the target domain as the source domain, while other domains with data are designated as auxiliary domains. Following this, we preprocess the original time-series data by converting it into time-frequency diagrams to construct the dataset for each domain. Thereafter, we conduct adversarial training of the generator and the domain index predictor, enabling the generator to produce data corresponding to a given operational condition domain index. Finally, we use the data generated by the generator for the target domain, in conjunction with the source domain data, to train a new fault diagnosis classifier, thereby enhancing the fault diagnosis performance for the target domain with no available data.

### 3.1. Data Preprocessing Module

The data preprocessing stage implements Short-Time Fourier Transform (STFT) [31] to convert temporal signals into time-frequency representations, thereby directing the model to acquire more precise feature representations. In practical applications, long-duration signals are divided into multiple short segments, which are then encoded separately into time-frequency diagrams, with each diagram serving as a training sample.

For a non-stationary signal x(t), given a narrow-width window function ω(t) that slides along the time axis, the STFT of the signal x(t) is defined as follows:

(1) $$STFT\left(\tau ,\omega \right)=\int_{-\infty }^{\infty }x\left(t\right)\omega \left(t-\tau \right)e^{-i\omega t}dt$$

where τ and ω represent the time and frequency axes, respectively.

### 3.2. Generator Module

Drawing inspiration from the concept of conditional generative adversarial networks [32], we utilize a conditional generator G to transform the source domain time-frequency diagram $X_{s}$ to a generated time-frequency diagram ${\tilde{X}}_{t}$ in the target domain.

The time-frequency diagram $X_{s}$ and the fault category $Y_{s}$ are input into the generator after encoding and concatenation to ensure the separability of different fault categories in the generated data. Therefore, the domain translation function is formalized as follows:

(2) $${\tilde{X}}_{t}=G\left(X_{s},Y_{s}\right)$$

The generator *G* is structured with a convolutional-deconvolutional architecture, as detailed in Table 2. Specifically, the generator’s architecture consists of an initial convolutional layer with a stride of 1, followed by two down-sampling convolutional layers, each with a stride of 2. The network further includes six residual blocks. For up-sampling, the architecture incorporates two transposed convolutional layers, each with a stride of 2, concluding with a final convolutional layer that operates with a stride of 1. The fault category is encoded as a one-hot vector, whose length is equivalent to the total number of fault categories. During forward propagation, the one-hot vector is initially spatially expanded and then integrated with the time-frequency diagram, thereby forming the input for the generator G.

### 3.3. Domain Index Predictor Module

Domain indices that are numerically proximate indicate analogous operational relationships. Consequently, minor variations in the domain index u should correspond to subtle alterations in the encoding. Thus, instead of classifying the encoding into categorical domains, our domain index predictor D regresses the domain index. Therefore, the domain index regression function is defined as

(3) $$U_{k}=D\left(X_{k}\right)$$

The domain index predictor D has a convolutional structure, as shown in Table 3, and consists of four convolutional layers with stride 2, three fully connected layers, and a Softplus activation function.

### 3.4. Fault Diagnosis Classifier Module

The classifier C can extract fault features from the time-frequency diagram data and then provide fault diagnosis results. The architecture of C is similar to that of D, with the distinction that the output of the final layer is a probability distribution of the classification results, and the classification loss is computed using the cross-entropy function. Therefore, the fault diagnosis classifier is defined as

(4) $$Y_{k}=C\left(X_{k}\right)$$

### 3.5. Optimization Objectives

#### 3.5.1. Optimization Objectives of Generator

The objective of the generator is to generate data that closely approximates the operational parameters of the target domain while ensuring the generated categories are separable and authentic. The loss function of the generator comprises three parts: domain index loss, classification loss, and reconstruction loss.

**Domain Index loss.** First, to render the generated data as closely aligned as possible with the operational conditions of the target domain, we utilize a domain index loss. This ensures that the data produced by the generator is predicted to the domain index of the target domain by the domain index predictor. The domain index loss $L_{d}$ is the $L_{2}$ loss between the predicted domain index and target domain index:

(5) $$L_{d}={\left\|D\left({\tilde{X}}_{t}\right)-u_{t}\right\|}_{2}^{2}$$

where ${\tilde{X}}_{t}$ is the generated data, and $u_{t}$ is the domain index of the target domain.

**Classification loss.** Secondly, in order to preserve the consistency of category labels, we necessitate that the synthetic data ${\tilde{X}}_{t}$ be assigned to the same category as the original data $X_{s}$.

(6) $$L_{c}=L_{CE}\left({C(\tilde{X}}_{t}),Y_{s}\right)$$

where $L_{CE}$ denotes cross-entropy loss, ${\tilde{X}}_{t}$ is the synthetic data, and $Y_{s}$ is the category label of the source domain.

**Reconstruction loss.** Finally, to guarantee authenticity, we apply a reconstruction loss to the generator. The reconstruction loss is an $L_{1}$ loss, which serves to quantify the difference between the generated samples and the source samples, thereby ensuring that the source samples are altered as minimally as possible during the data generation process to guarantee the authenticity of the generated data.

(7) $$L_{r}={\left\|{\tilde{X}}_{t}-X_{s}\right\|}_{1}$$

**Generator training.** The complete objective function for G is a weighted sum of Equations (5)–(7),

(8) $$L_{G}=L_{d}+L_{c}+L_{r}$$

#### 3.5.2. Optimization Objectives of Domain Index Predictor

The domain index predictor D is capable of predicting the corresponding domain index from time-frequency diagrams from different domains. The loss function of the domain index predictor is divided into two parts: domain index regression loss $L_{d1}$ and adversarial domain index regression loss $L_{d2}$. $L_{d1}$ ensures the effectiveness of D on known data, while $L_{d2}$ encourages the generator to predict the domain index of the generated sample ${\tilde{X}}_{t}$ as the source domain index.

Generator G and predictor D engage in adversarial training, where G attempts to make D predict the domain index of the generated data as the target domain index. Meanwhile, D attempts to predict the generated samples ${\tilde{X}}_{t}$ as the source domain index $u_{s}$.

**Domain Index Regression loss.** Firstly, $L_{d1}$ employs an $L_{2}$ loss to ensure accurate prediction of D for data from the source and auxiliary domains.

(9) $$\begin{array}{c} L_{d1}=\lambda_{s}{\left\|D\left(X_{s}\right)-u_{s}\right\|}_{2}^{2}+\lambda_{a_{1}}{\left\|D\left(X_{a}^{1}\right)-u_{a}^{1}\right\|}_{2}^{2}\\ +\lambda_{a_{2}}{\left\|D\left(X_{a}^{2}\right)-u_{a}^{2}\right\|}_{2}^{2}+\ldots +\lambda_{a_{Ma}}{\left\|D\left(X_{a}^{M_{a}}\right)-u_{a}^{M_{a}}\right\|}_{2}^{2} \end{array}$$

where $X_{a}^{n}$ is the frequency diagram of the nth auxiliary domain and $u_{a}^{n}$ is the domain index of the nth auxiliary domain. λ is a hyperparameter representing the weights of different domains, usually 1, which can be set according to the proportion of sample sizes in different domains. $M_{a}$ is the number of auxiliary domains.

**Adversarial Domain Index Regression loss.** Subsequently, $L_{d2}$ motivates D to predict the domain index of the generated samples as the source domain index.

(10) $$L_{d2}={\left\|D\left({\tilde{X}}_{t}\right)-u_{s}\right\|}_{2}^{2}$$

**Predictor training.** The complete objective function for D is a weighted sum of Equations (9) and (10),

(11) $$L_{D}=L_{d1}+\lambda_{d2}L_{d2}$$

where $\lambda_{d2}$ is a weighting hyperparameter.

#### 3.5.3. Optimization Objectives of Fault Diagnosis Classifier

The fault diagnosis classifier C is trained on both the source domain data $X_{s}$ and the generated data ${\tilde{X}}_{t}$, and its loss function is

(12) $$L_{C}=L_{CE}\left(C(X_{s}),Y_{s}\right)+L_{CE}\left(C({\tilde{X}}_{t}),Y_{s}\right)$$

where $L_{CE}$ is the cross-entropy loss.

#### 3.5.4. Implementation of Multiple Domain Index Predictors

In a standard adversarial framework, a single-domain index predictor serves as the critic to guide the generator. However, relying on a single regressor can lead to instability. The generator may “overfit” to the specific decision boundary of one predictor, finding adversarial examples that satisfy the numerical index constraint without truly acquiring the underlying physical characteristics of the target domain. This phenomenon is analogous to “mode collapse” in conditional GANs.

To address this, we propose a multiple-domain index predictor strategy. We construct a set of K predictors, $D=\{D_{1},D_{2},\ldots ,D_{K}\}$, having identical architectures but distinct random initializations. This ensemble acts as a robust regularization mechanism. During adversarial training, the generator must synthesize features that are consistently mapped to the target index $u_{t}$ by the entire consensus of predictors. This constraint forces the generator to learn a smooth and generalized mapping from the domain index space to the feature space, rather than exploiting the blind spots of a single network.

Consequently, the adversarial loss for the generator is computed as an adaptive weighted aggregation of the feedback from all K predictors:

(13) $$L_{d}=\sum_{k=1}^{K}\frac{{\left\|D_{k}\left({\tilde{X}}_{t}\right)-u_{t}\right\|}_{2}^{2}}{L_{d1}^{k}+\varepsilon }$$

where K is the number of domain index predictors and $L_{d1}^{k}$ is the domain index regression loss of the kth domain index predictor.

For numerical stability, the denominator in Equation (13) is written as $L_{d1}^{k}+\varepsilon$, where ε is a small positive constant, set to $1\times 10^{-6}$ in our implementation. This term prevents the adversarial objective from becoming unbounded when $L_{d1}^{k}$ is extremely small, while having a negligible effect when $L_{d1}^{k}$ is not close to zero.

### 3.6. Full Training Algorithm

The complete training procedure of the proposed WCIGDG framework is summarized in Algorithm 1, which proceeds in two sequential stages: the adversarial training stage and the fault diagnosis model training stage.

#### 3.6.1. Adversarial Training Stage

To ensure stability and effective domain alignment, the adversarial training process employs an alternating optimization strategy within each epoch, comprising two distinct phases:

**Phase 1: Critic and Classifier Optimization (Fix** G**, Update** C**,** $\left\{D_{K}\right\}$**).** In this phase, the generator G is frozen. We optimize the fault diagnosis classifier C and multiple domain index predictors $\left\{D_{K}\right\}$ to ensure they can accurately process both real and generated data.

Classifier Update: C is trained to minimize the cross-entropy loss on both real source data $X_{s}$ and generated data ${\tilde{X}}_{t}$ to maintain class separability.

Predictor Update: Each predictor $D_{K}$ is trained to accurately regress the domain indices of source data $X_{s}$ and auxiliary data $X_{a}$. Simultaneously, they are optimized to regress the generated data ${\tilde{X}}_{t}$ towards the source domain index $u_{s}$. This step ensures that the predictors serve as reliable “critics” that understand the physical manifold of the operating conditions.

**Phase 2: Generator Optimization (Fix** C**,** $\left\{D_{K}\right\}$**, Update** G**)**

In this phase, C and $\left\{D_{K}\right\}$ are frozen. G is updated to synthesize realistic features for the target domain. The generator has three concurrent objectives:

Reconstruction ($L_{r}$): Minimize the $L_{1}$ distance between generated and input features to preserve content information.

Semantic Consistency ($L_{c}$): Minimize the classification loss calculated by C to ensure the generated data retains the correct fault class information.

Adversarial domain index guidance ($L_{d}$): Minimize the target-index regression loss under the adaptive multi-predictor adversarial objective, thereby shifting generated features toward the target-domain manifold.

#### 3.6.2. Fault Diagnosis Model Training Stage

Upon completion of the adversarial training iterations, the optimized generator G is frozen. It is then utilized to synthesize a target domain dataset ${\tilde{X}}_{t}$ using source data as input. Finally, this synthetic data is combined with the original source domain data $X_{s}$ to train a new, robust classifier $\tilde{C}$ for fault diagnosis in the target domain.
**Algorithm 1:** WCIGDG**# Adversarial Training stage****Input**: source domain dataset $D^{s}$, multiple auxiliary domain datasets $D^{a}={\left\{D_{m}^{a}\right\}}_{m=1}^{M_{a}}$1: **for** epoch = 1 to epochs = *N*
**do**2: Forward propagation to generate ${\tilde{X}}_{t}=G(X_{s},Y_{s})$.3: Calculate $L_{C}$ and $L_{D}$
4: Backward propagation to update C and D
5: Calculate $L_{G}$
6: Backward propagation to update G
  **Return**: Trained generator G
**# Fault Diagnosis Model Training stage**  **Input**: source domain dataset $D^{s}$.1: Forward propagation to generate ${\tilde{X}}_{t}=G(X_{s},Y_{s})$.2: **for** epoch = 1 to epochs = *N* **do**3: Calculate $L_{C}$ based on the classification results of generated data ${\tilde{X}}_{t}$ and real data from source domain $X_{s}$.4: Backward propagation to update $\tilde{C}$
  **Return**: Trained classifier $\tilde{C}$


### 3.7. Implementation Details and Hyperparameter Settings

To ensure the reproducibility of our experiments, we detail the specific implementation parameters and hyperparameter selections used in this study.

The input time-frequency diagrams were resized to 64×64. We utilized the RMSprop optimizer for both training stages: the adversarial training stage employed a learning rate of 0.0002 for 30 epochs, while the subsequent fault diagnosis model training stage used an initial learning rate of 0.001 for 300 epochs, which was decayed by a factor of 0.1 at the 150th and 250th epochs using a step scheduler. The batch size was set to 64. Regarding the loss function weights, the domain weights in the predictor loss function (Equation (9)) were set equally to λ=1, and the weighting factor $\lambda_{d2}$ in the adversarial predictor loss (Equation (11)) was set to 0.1 based on grid search results to balance regression accuracy and adversarial resistance. The three components of the generator loss (Equation (8)) were weighted equally (coefficients set to 1.0), and the number of domain index predictors (Equation (13)) was set to K=20 based on grid search results.

## 4. Experimental Study

### 4.1. Dataset

In this article, we validate and analyze our proposed method using two publicly accessible datasets.

#### 4.1.1. Case Western Reserve University (CWRU) Dataset

The CWRU bearing dataset, a well-established benchmark in the domain of bearing fault diagnosis [33], comprises four distinct bearing health conditions: normal, outer-race fault, inner-race fault, and rolling-element fault. By considering three varying fault sizes for each fault type, the dataset is classified into ten classes overall, with each class containing an equal number of samples. The CWRU dataset comprises four distinct rotational speeds and is sampled at a frequency of 12 kHz.

We acknowledge that CWRU is a widely used but relatively idealized bearing benchmark and has known limitations, including simplified operating conditions and signal characteristics that may not fully represent industrial machinery. It is retained here because it enables comparison with extensive prior fault diagnosis studies and provides multiple rotational-speed conditions required by the indexed domain setting. The more challenging WT planetary gearbox dataset is therefore used as primary empirical evidence, and the conclusions are not based solely on CWRU.

#### 4.1.2. WT-Planetary Gearbox Dataset

The WT dataset [34] is an open-source planetary gearbox dataset provided by Beijing University of Technology, with data collected from a wind turbine system test rig. It includes five types of gear health conditions: normal, gear tooth breakage, gear wear, root crack, and missing tooth, with each class containing an equal number of samples. The WT dataset comprises eight rotational speeds and is sampled at a frequency of 48 kHz.

#### 4.1.3. Domain Generalization Task Setup

In practical applications, our method selects the source domain with the domain index closest to the target domain from the available source domains. To validate the superiority of the proposed method under various potential scenarios in experiments, we exhaustively evaluated all possible source-target domain combinations within the dataset (with the remaining domains serving as unlabeled auxiliary domains). These combinations were categorized into tasks of varying difficulty levels based on differences in rotational speed.

Denoting the working conditions corresponding to the four rotational speeds {1797 rpm,1772 rpm,1750 rpm,1730 rpm} in the CWRU dataset as domains {cw0,cw1,cw2,cw3}, we constructed 12 domain generalization tasks within the CWRU dataset, as presented in Table 4. These tasks were grouped into three difficulty levels: {CW0,CW1,CW2}.

**Figure 2:**
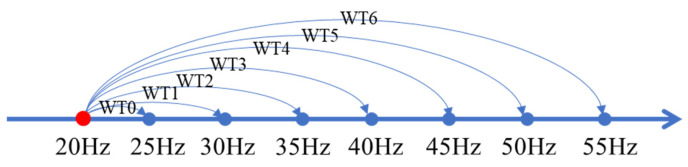
Examples of domain generalization tasks with different difficulties.

Similarly, denoting the working conditions corresponding to the eight rotational speeds $\left\{20\;Hz,\;25Hz,\;30\;Hz,\;35\;Hz,\;40\;Hz,\;45\;Hz,\;50\;Hz,\;55\;Hz\right\}$ in the WT dataset as domains {wt0,wt1,wt2,wt3,wt4,wt5,wt6,wt7}, we constructed 56 domain generalization tasks within the WT dataset, as shown in Table 5. These tasks were grouped into seven difficulty levels: {WT0,WT1,WT2,WT3,WT4,WT5,WT6}. Figure 2 illustrates examples of tasks at different difficulty levels using wt0 as the source domain.

To avoid training-test leakage, the target domain in each transfer task was excluded from generator training, domain index predictor training, classifier training, and baseline training. Task groups such as WT5 are used only for reporting task difficulty; each source-target pair in a group is trained and tested independently. For example, wt6–wt0, wt0–wt6, wt7–wt1, and wt1–wt7 are four separate experiments, and the target-domain samples in each experiment are used only for final testing.

### 4.2. Experimental Setup

In the data preprocessing phase, each raw vibration signal was segmented into non-overlapping samples of 1024 points. The STFT was computed using the SciPy implementation with a Hann window, a 256-sample window length, a 250-sample overlap (97.7% overlap ratio), and 256 FFT points. The sampling frequency was set according to the original sampling rate of each dataset: 12 kHz for the CWRU dataset and 48 kHz for the WT dataset. For real-valued vibration signals, the one-sided STFT magnitude spectrum was retained, corresponding to frequency ranges of 0–6 kHz for CWRU and 0–24 kHz for WT. The resulting STFT magnitude maps were resized to 64 × 64 using the zoom interpolation function in SciPy.

Rotational speed was used as the domain index in both CWRU and WT datasets because the available working-condition changes in these datasets are speed changes. To ensure the physical consistency of the domain manifold, we employed Min-Max normalization to scale the domain index to the range [0, 1]. All data were used in the CWRU dataset. Due to the large amount of data in the WT dataset, the first 500,000 data points of each WT signal file were used. To ensure the validity of the experiments and the fairness of comparison, all experiments were conducted under the same experimental environment: Windows 11, an NVIDIA GeForce RTX 5090 series GPU (NVIDIA Corporation, Santa Clara, CA, USA), and PyTorch 2.9.1.

All methods were evaluated under the same data-availability condition. For each transfer task, target-domain samples were excluded from model training. For DANN and CIDA, which are originally domain-adaptation methods, target-domain access was removed; they were trained only with the source domain and non-target auxiliary domains, so the comparison follows the domain generalization setting used for WCIGDG.

The following baseline methods are compared in this study.

**Convolutional Neural Network (CNN)**: This method directly employs the CNN model, which has been trained in the source domain for application in the target domain.

**Domain-Adversarial Network (DANN)** [4]: This method employs the principle of adversarial training [35] across multiple source domains to identify a shared feature representation, with the objective of constructing a more robust diagnostic model.

**Continuously Indexed Domain Adaptation (CIDA)** [18]: This method models multiple domains using continuous domain indices and employs a domain index predictor for adversarial training to discover shared feature representations. As CIDA is designed for domain adaptation tasks, it requires target domain data for training. In the context of domain generalization tasks, we simplify this approach by training with data from multiple source domains only.

**Domain generalization with mixstyle (Mixstyle)** [36]: A parameter-free module that mixes feature statistics during training to improve domain generalization without extra data or model capacity.

**Mutual-assistance semisupervised domain generalization network (MSDGN)** [15]: This method leverages pseudo-labeling of unlabeled data, coupled with an entropy-based sample refinement mechanism, to boost sample quality.

**Semi-Supervised Dynamic Generalization Network (SDGN)** [14]: A recently proposed method (2025) that utilizes a dynamic feature extractor with a dual feature enhancement strategy to handle unseen working conditions.

### 4.3. Results and Analysis

#### 4.3.1. Diagnosis Results of Domain Generalization Tasks

The quantitative diagnosis results for the CWRU and WT datasets are presented in Table 6 (Accuracy on CWRU), Table 7 (Accuracy on WT), Table 8 (F1-Score on CWRU), and Table 9 (F1-Score on WT). In Table 6 and all subsequent tables, bold values indicate the best result in each column.

**Figure 3:**
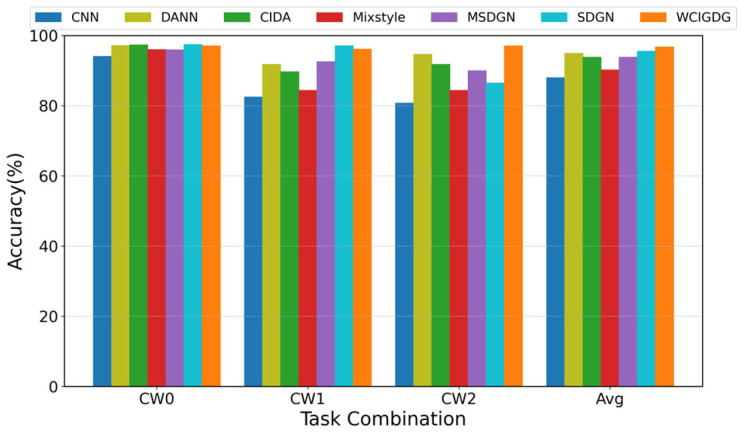
Grouped bar chart of accuracy by task group on the CWRU bearing dataset.

**Figure 4:**
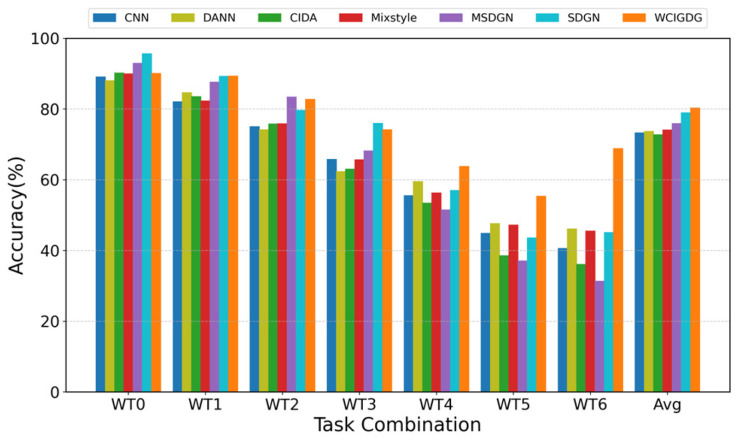
Grouped bar chart of accuracy by task group on the WT planetary gearbox dataset.

**Overall Performance Superiority:** As observed in Figure 3 and Figure 4, the proposed WCIGDG method achieves the highest average performance on both datasets.

On the CWRU dataset, WCIGDG attains an overall average accuracy of 96.74% and an F1-Score of 96.44%. This outperforms the state-of-the-art SDGN method, which achieves 95.52% accuracy and 93.72% F1-Score.

On the WT dataset, which features significant speed variations, WCIGDG demonstrates robust generalization with an average accuracy of 80.28% and an F1-Score of 79.59%. In comparison, SDGN achieves 78.95% accuracy and 77.66% F1-Score, while MSDGN lags with 75.93% accuracy.

**Figure 5:**
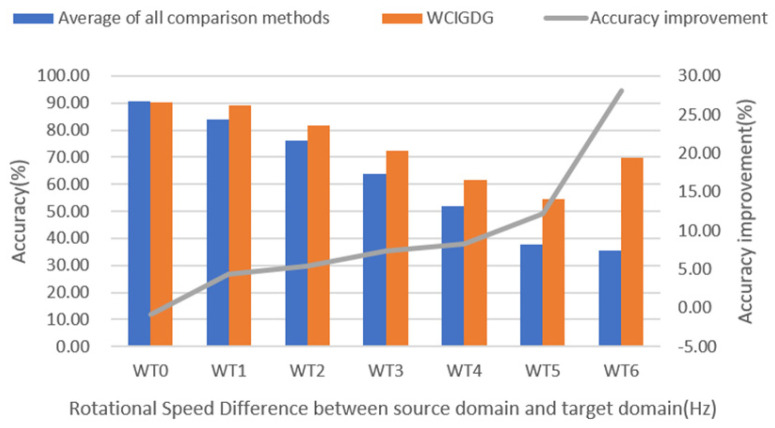
The accuracy improvement achieved by WCIGDG over the average of all comparison methods.

**Robustness Under Large Domain Shifts:** As observed in Figure 5, the performance advantage of WCIGDG becomes more pronounced as the domain discrepancy increases.

In the WT dataset, as the speed difference between source and target domains widens (e.g., tasks in group WT6 involving a 35 Hz difference), traditional feature alignment methods like CIDA and Mixstyle suffer noticeable performance degradation.

Conversely, WCIGDG maintains satisfactory performance in these challenging scenarios. For instance, in the WT6 task group, WCIGDG achieves an average accuracy of 68.85%, significantly surpassing CIDA (36.07%) and MSDGN (31.35%). This indicates that the generative strategy guided by the domain index effectively synthesizes features close to the unseen target distribution, thereby bridging large domain gaps.

**Performance Analysis on Low-Discrepancy Tasks:** It is worth noting that in tasks with minimal speed variations (e.g., CW0 tasks in CWRU with ~22 rpm difference), methods like SDGN (97.49%) and CIDA (97.32%) perform slightly better than WCIGDG (97.06%). This is likely because, in simple scenarios with negligible domain shifts, the additional data variance introduced by the generative process may slightly outweigh the benefits of domain alignment. However, this marginal trade-off in simple tasks yields substantial gains in more complex and realistic scenarios (e.g., CW2 and WT6 tasks), where WCIGDG consistently outperforms all baselines.

#### 4.3.2. Confusion Matrix Analysis

To investigate failure modes under significant domain shifts, the confusion matrices for tasks cw3–cw0 (CWRU) and wt1–wt3 (WT) are visualized and analyzed.

**Figure 6:**
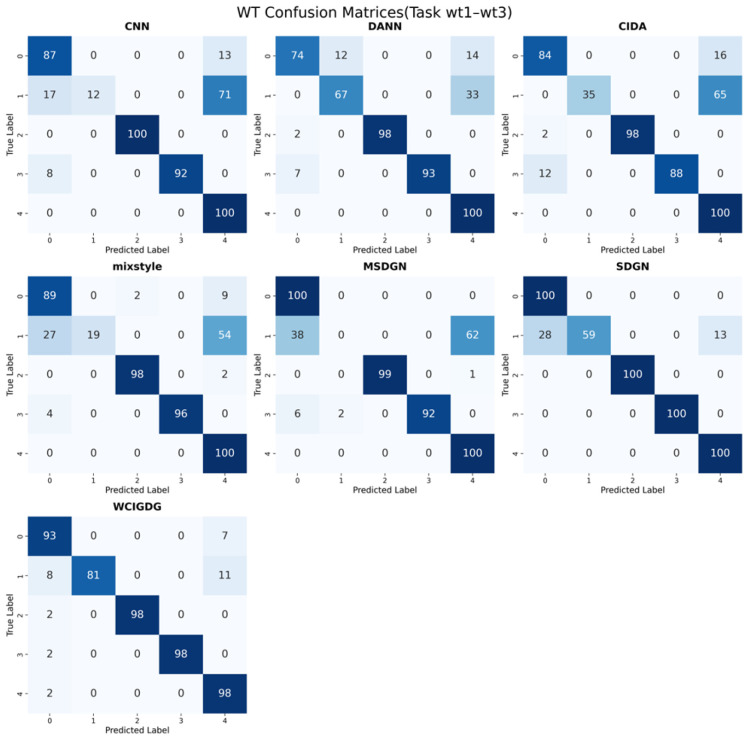
Confusion matrices on the WT planetary gearbox dataset (task wt1–wt3). Darker blue cells indicate higher percentages.

As shown in Figure 6, in the challenging WT task (wt1–wt3), baseline methods exhibit severe misclassification on specific fault categories. For instance, Class 1 is heavily confused with Class 4 by CIDA and with Class 0 by SDGN. In contrast, the proposed WCIGDG significantly improves Class 1 accuracy.

**Figure 7:**
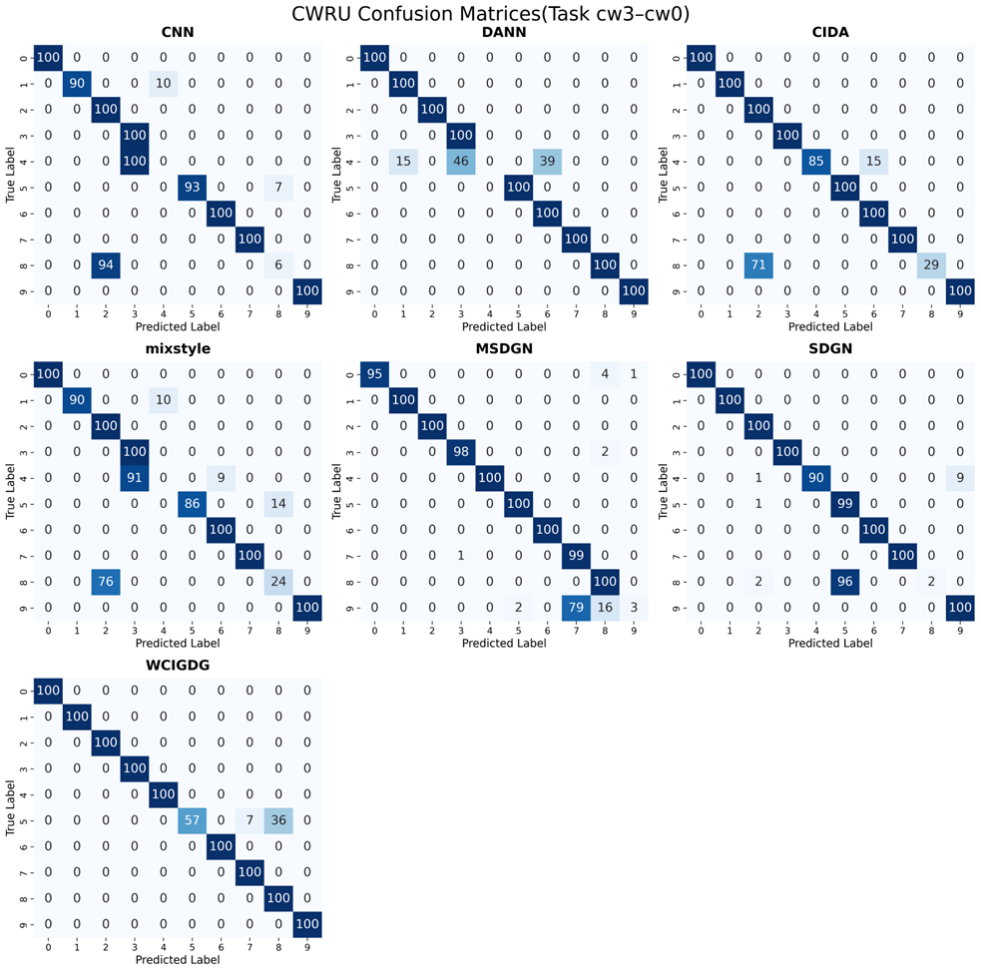
Confusion matrices on the CWRU bearing dataset (task cw3–cw0). Darker blue cells indicate higher percentages.

Similarly, as shown in Figure 7, in the CWRU task (cw3–cw0), while baselines generally perform well, some of them fail to accurately identify Class 4; WCIGDG completely resolves this ambiguity.

These results demonstrate that WCIGDG establishes more robust decision boundaries for fault categories that are easily confused under unseen working conditions.

#### 4.3.3. Ablation Experiments

Ablation studies evaluate the contributions of domain index guidance (M2), adversarial training (M3), and multiple predictors (WCIGDG). As shown in Table 10, the ablation setup systematically removes each component to assess its impact. Results in Table 11 (CWRU) and Table 12 (WT) show a consistent performance progression. Notably, on the complex WT dataset, the multiple predictor strategy is critical, boosting accuracy from 75.12% (M3) to 80.28% (WCIGDG), proving its necessity for capturing diverse domain characteristics.

#### 4.3.4. Optimal Hyperparameter Determination

**Figure 8:**
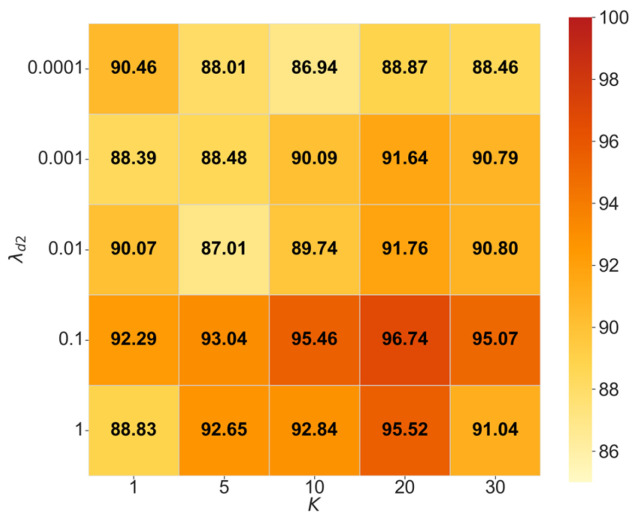
Average accuracy (%) under different hyperparameter settings.

In our method, the primary hyperparameters include $\lambda_{a_{i}}$ (Equation (9)) controlling the weighting of samples from different domains, $\lambda_{d2}$ (Equation (11)) controlling the adversarial strength, and K (Equation (13)) controlling the number of domain index predictors. Since the sample sizes across all domains were identical in the experiments, $\lambda_{a_{i}}$ was uniformly set to 1. Hyperparameters $\lambda_{d2}$ and K were selected by grid search on one of the CWRU tasks, whereas Figure 8 reports the average accuracy across all CWRU tasks for sensitivity analysis. The values of $\lambda_{d2}$ and K were searched over the sets {0.0001, 0.001, 0.01, 0.1, 1} and {1, 5, 10, 20, 30}, respectively. The results indicate that the optimal values for $\lambda_{d2}$ and K are 0.1 and 20, respectively.

The optimal hyperparameters selected on the CWRU tuning task were fixed and directly transferred to the WT experiments. No separate grid search was conducted on WT, which avoids dataset-specific tuning and provides a stricter cross-dataset evaluation protocol.

## 5. Analysis and Discussion

### 5.1. Domain Index Normalization and Physical Justification

To investigate the impact of domain index preprocessing on model generalization and to justify our choice of linear scaling, we evaluated distinct normalization strategies and analyzed the underlying physical relationships driving the domain shift.

#### 5.1.1. Comparison of Normalization Schemes

We compared three normalization strategies on the WT dataset using the transfer task wt3–wt0 (transferring from 35 Hz to 20 Hz). The strategies are defined as follows:

Min-Max Normalization: This assumes a linear correspondence between the index value and the domain shift.

(14) $$\tilde{v}=\frac{v-v_{min}}{v_{max}-v_{min}}$$

Z-score Standardization: This scales the index based on the statistical distribution of the domain index.

(15) $$\tilde{v}=\frac{v-\mu }{\sigma }$$

Logarithmic Transformation: This applies a non-linear smoothing prior to normalization.

(16) $$\tilde{v}=MinMax\left(\ln\left(v\right)\right)$$

The results in Table 13 demonstrate that Min-Max normalization achieves superior performance with an accuracy of 95.08%. In contrast, Z-score standardization yields a lower accuracy of 89.75%, and the logarithmic transformation significantly degrades performance to 79.10%.

#### 5.1.2. Justification Based on Domain Distribution Shifts

**Figure 9:**
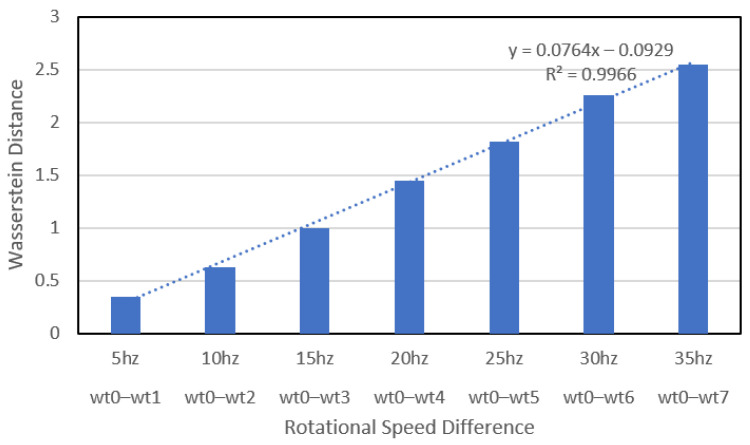
Relationship between rotational speed gap and distribution shift. Bars show the distances and the dotted line shows the linear fit.

To explain the superiority of linear normalization, we quantitatively analyzed the relationship between the Domain Index Difference (the physical rotational speed gap Δv) and the Feature Distribution Shift (measured by the Wasserstein distance $W_{D}$, between the source and target domain features). As illustrated in Figure 9 and Table 14, there exists a clear linear positive correlation between the two variables.

When the speed difference is small (e.g., 5 Hz), the domain shift is minimal ($W_{D}\approx 0.35$).

As the speed difference increases to 35 Hz, the domain shift scales proportionally ($W_{D}\approx 2.55$).

The linear growth of the Wasserstein distance in this experiment suggests that the domain shift accumulates approximately proportionally with the rotational-speed difference. This result supports using Min-Max normalization for the present benchmark setting because it preserves the relative spacing of speed conditions. For other types of domain indices, such as nonlinear pressure, load coupling, resonance, looseness, sensor-position changes, or complex contact dynamics, the normalization strategy should be selected according to the corresponding physical relationship; nonlinear transformations, multidimensional indices, or learned index mappings may be more suitable.

#### 5.1.3. Strategy for Target Index Estimations

In practical deployment scenarios, the target operating parameters may not be precisely known. Provided that unlabeled data from the target domain is available, and the domain index satisfies the monotonic distribution shift assumption—meaning the magnitude of the domain shift typically scales with the difference in the operating condition—the unknown target index can be inversely inferred.

Specifically, we compute the $W_{D}$ between the incoming unlabeled target data and the stored source domains. Assuming the linear model $W_{D}\approx k\cdot \left|u_{t}-u_{s}\right|+b$ holds for the specific operating parameter, the estimation is performed via linear extrapolation.

While this estimation relies on the validity of the linear assumption for the specific physical parameter, our robustness analysis in Section 5.3 demonstrates that the WCIGDG method remains effective even if the estimation carries reasonable error. This makes the strategy a viable solution for operating conditions that exhibit systematic distributional shifts.

This strategy is an optional deployment extension for cases where the target operating parameter is unavailable but unlabeled monitoring data can be collected. It is not used in the training, validation, hyperparameter selection, or experimental evaluation reported in this study. All reported target-data-free results rely only on the known target operating parameter and do not use target-domain samples during training.

### 5.2. Computational Efficiency Analysis

To assess the practical deployability of the WCIGDG method, we conducted a comprehensive analysis of its computational costs. All experiments were performed on a workstation running Windows 11 with an NVIDIA GeForce RTX 5090 series GPU, and the method was implemented in PyTorch 2.9.1.

#### 5.2.1. Training Time Comparison

Table 15 reports the average training duration per transfer task. The proposed WCIGDG (configured with K = 20 domain index predictors) requires 188.04 s on the CWRU dataset and 395.55 s on the WT dataset. While the inclusion of multiple domain index predictors introduces additional overhead compared to a basic CNN, WCIGDG maintains high efficiency relative to state-of-the-art domain generalization frameworks. Specifically, it outperforms MSDGN on both datasets (341.89 s on CWRU; 448.44 s on WT) and achieves speeds comparable to or faster than SDGN and CIDA. This demonstrates that WCIGDG effectively trades a moderate increase in computation for significant gains in generalization performance without becoming a bottleneck.

#### 5.2.2. Scalability of the Number of Predictors (K)

**Figure 10:**
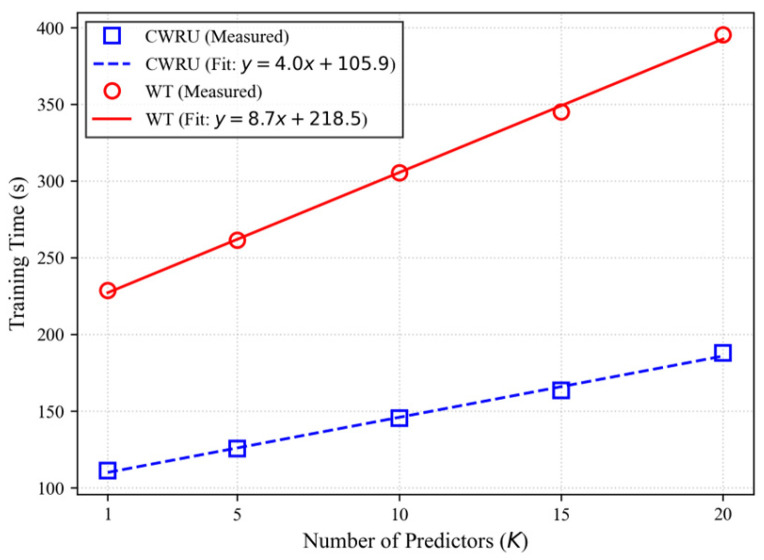
Scalability of the Number of Predictors (K).

A key hyperparameter in our framework is K, the number of domain index predictors. We investigated how increasing K affects computational load. As shown in Figure 10, the training time exhibits a strong linear correlation with K. Regression analysis indicates that adding one predictor increases training time by approximately 4.0 s for CWRU and 8.7 s for WT. This linear scaling property is crucial for industrial feasibility, as it ensures that the computational cost remains predictable.

### 5.3. Robustness Analysis Against Domain Index Noise

In industrial deployment, precise target domain indices are often unavailable due to measurement noise or estimation errors. To evaluate the robustness of WCIGDG against such perturbations, we conducted experiments on the WT dataset using the transfer task wt3–wt0. We simulated index estimation errors by injecting Gaussian noise δ into the true domain index u. The model was then conditioned on the perturbed index $u^{\prime }=u+\;\delta$, where $\delta \sim N(0,\sigma^{2})$. The noise intensity, represented by the standard deviation σ, was varied from 0.05 Hz to 5 Hz. Given that the interval between adjacent source domain indices in this dataset is approximately 5 Hz, these noise levels correspond to perturbations ranging from 1% to 100% of the domain interval.

The results, presented in Table 16, indicate that the method can exhibit resilience to index noise.

Minor Perturbations: Under small noise (σ=0.05 Hz), the accuracy slightly improved to 95.90%. This observation implies that minor perturbations might help the model generalize better in certain cases, possibly acting as a form of noise injection regularization.

Wide Tolerance: In this experiment, the model maintained stable performance with accuracy exceeding 90.57% even as σ increased to 2.5 Hz. This suggests that the generator is likely learning a smooth manifold of features with respect to the domain index.

Failure Boundary: A significant performance drop (to 79.51%) was observed when σ reached 5 Hz. At this level, the noise magnitude equals the distance between distinct operating conditions, which likely causes the model to misinterpret the domain semantics.

The experimental results suggest that WCIGDG can remain effective even with estimated indices, provided the estimation error is kept within a reasonable range relative to the operating condition interval.

### 5.4. Domain Index Generalizability and Extensions

#### 5.4.1. Applicability to Other Operating Parameters

While this study utilizes rotational speed as the representative domain index, the WCIGDG framework is theoretically agnostic to the specific physical meaning of the index. It is applicable to other continuous operating parameters, such as load torque, temperature, or hydraulic pressure, provided they satisfy the monotonic distribution shift assumption. As demonstrated in our Wasserstein distance analysis, successful indexing requires that the magnitude of the domain shift (distributional discrepancy) exhibits a strong positive correlation—typically monotonic—with the distance in the domain index space.

Under such conditions, the adversarial training mechanism can effectively learn the mapping from the parameter space to the feature distribution space, regardless of whether the parameter is speed or load. Conversely, for parameters that induce chaotic or non-systematic changes in signal characteristics, the continuity assumption may not hold, limiting the method’s effectiveness.

#### 5.4.2. Interpolation and Extrapolation of Domain Indices

To examine the behavior when the target domain index lies inside or outside the range of available indices, we conducted an additional experiment on the WT transfer task wt2–wt6. The source and target domains were kept fixed, while the auxiliary domains available to train the domain index predictor were progressively changed. When wt0–wt5 and wt7 were available, the target domain wt6 was an interpolation case. When only wt0–wt5 were available, wt6 became an extrapolation case, and removing wt5, wt4, and wt3 further increased the extrapolation distance.

The results, presented in Table 17, show a clear difference between interpolation and extrapolation. The interpolation setting provides the best performance, whereas extrapolation accuracy and F1-Score decrease as the target index moves farther beyond the maximum available index. Nevertheless, all WCIGDG extrapolation settings remain above the CNN baseline, indicating that the indexed generation mechanism can still provide useful guidance under moderate extrapolation, although auxiliary conditions close to the target index are beneficial for stable generalization.

#### 5.4.3. Extension to Multidimensional Domain Indices

Industrial machinery often operates under complex conditions defined by multiple simultaneous variables (e.g., a specific combination of speed and load). The proposed framework naturally extends to Multidimensional Domain Indices. In such scenarios, the scalar index u is replaced by a vector $u={\left[u_{speed},\;u_{load}\right]}^{T}$.

The domain index predictor D is modified to function as a multivariate regressor, outputting a vector prediction $\hat{u}$. The loss function is updated to minimize the Euclidean distance between the predicted and ground-truth vectors.

Although the current experimental validation is constrained to unidimensional indices due to the lack of public benchmarks with simultaneous, granular variations in multiple conditions, the theoretical foundation of WCIGDG supports high-dimensional index spaces.

Validation under simultaneous multi-variable changes, such as coupled speed-load variation, remains an important future work direction. At present, public data-free target-domain benchmarks with sufficiently granular multidimensional operating conditions are limited; establishing such datasets would be valuable for future research on indexed domain generalization in industrial fault diagnosis.

### 5.5. Deployment Prerequisites and Applicable Scenarios

To guide practitioners in applying WCIGDG, we analyze the critical prerequisites and suitable scenarios for deployment. Unlike traditional methods, WCIGDG relies on modeling the continuous evolution of faults. Therefore, its deployment is subject to specific data and physical constraints.

#### 5.5.1. Deployment Requirements

Successful deployment requires meeting three key conditions:

Multi-Condition Data: The method requires data from at least two distinct operating conditions, including at least one labeled source domain and one or more non-target auxiliary domains with known indices. The auxiliary domains may be unlabeled and are mainly used to train the domain-index predictor. This Source Diversity is essential for the domain index predictor to regress the relationship between the condition parameters and feature representations. The method is not applicable if the historical database contains data from only a single, static operating point.

Validity of Domain Index (Monotonicity): The selected domain index must physically govern the domain shift. As demonstrated in our Wasserstein distance analysis, there must be a systematic—typically monotonic or linear—correlation between the difference in the domain index and the distributional shift in the feature space.

Availability of Target Information: For the target domain, the system requires either the explicit value of the operating parameter (via sensors) or sufficient unlabeled data to estimate it using the distribution alignment strategy described in Section 5.1.

#### 5.5.2. Applicable Industrial Scenarios

Based on these requirements, WCIGDG is best suited for the following scenarios:

Variable-Speed/Load Machinery: Equipment like wind turbines, centrifugal pumps, or electric vehicles where operating conditions (speed and torque) vary continuously and are monitored by SCADA systems.

Sparse Data Regimes: Scenarios where it is impractical to collect fault data for every possible operating set point, but fault data exist for a few discrete set points.

Conversely, the method is less effective for systems subject to chaotic or non-systematic shifts, where changes in operating conditions cause abrupt, non-linear changes in failure modes that cannot be modeled by a continuous manifold.

## 6. Conclusions

This article proposes the WCIGDG model for cross-domain fault diagnosis under unseen conditions based on the working-condition domain indexing. The model comprises modules for STFT data preprocessing, domain index predictor, data generator, and fault diagnosis classifier. We validate the effectiveness and advantages of the WCIGDG model through 68 domain generalization tasks across two datasets from multiple perspectives. The experimental analysis yields three principal findings:

(1) In domain generalization across indexed domains, the WCIGDG model achieves higher fault diagnosis accuracy compared to other models, particularly demonstrating enhanced performance in more challenging domain generalization tasks.
(2) The empirical results validate the core premise that variations in operating conditions—specifically rotational speed as validated in this study—induce systematic rather than chaotic distribution shifts.
(3) The method exhibits strong robustness and reasonable computational efficiency. Quantitative analysis shows that the model maintains stable diagnostic performance even when domain indices are subject to estimation noise, and it achieves these generalization gains with a training overhead comparable to other leading methods, ensuring its feasibility for industrial deployment.

In summary, the proposed WCIGDG model can effectively enhance the generalization capability for domains without data under known working conditions, addressing the domain shift issue caused by different working conditions. Despite these advancements, the current framework relies on the availability of multi-condition historical data and assumes monotonic distribution shifts. Future work will extend the domain indexing mechanism from unidimensional parameters to multidimensional vectors (e.g., simultaneous speed and load variations) to accommodate more complex, non-linear industrial environments.

## Figures and Tables

**Table 1:** Notation table.

Symbol	Meaning	Data Availability/Role
$D^{s}$	Source domain with labeled data and known domain index	Available for training
$D^{a}$	Auxiliary domains with unlabeled data and known domain indices	Available for predictor training
$D^{t}$	Target domain at a specified working-condition index	Unavailable during training
$X_{s}$, $Y_{s}$	Source domain samples and labels	Available for training
$X_{a}^{m}$	Samples from the m-th auxiliary domain	Available for predictor training
$X_{t}$, $Y_{t}$	Real target domain samples and labels	Not used during training
$u_{s}$, $u_{a}^{m}$, $u_{t}$	Domain indices of source, m-th auxiliary, and target domains	Known working-condition parameters
G, D, C	Generator, domain-index predictor, and fault diagnosis classifier	Model components

**Table 2:** Structural parameters of the generator.

Layer Name	Kernel Size/Node	Kernel Number	Stride	Operation and Activation
Conv	7	64	1	IN, RELU
Conv	4	128	2	IN, RELU
Conv	4	256	2	IN, RELU
Residual block × 6	3	256	1	IN, RELU
Deconv	4	128	2	IN, RELU
Deconv	4	64	2	IN, RELU
Conv	7	1	1	IN, RELU, Tanh

**Table 3:** Structural parameters of the domain index predictor.

Layer Name	Kernel Size/Node	Kernel Number	Stride	Operation and Activation
Conv	4	32	2	IN, RELU
Conv	4	64	2	IN, RELU
Conv	4	128	2	IN, RELU
Conv	4	256	2	IN, RELU
AMP	4	–	–	-
FC	256	–	–	RELU, Dropout
FC	256	–	–	RELU, Dropout
FC	1	–	–	Softplus

**Table 4:** Domain generalization tasks of the CWRU dataset.

Task Group	Rotational-Speed Difference (Average)	Domain Generalization Tasks
CW0	22.3 rpm	cw1–cw0, cw0–cw1, cw1–cw2, cw2–cw1, cw2–cw3, cw3–cw2,
CW1	44.5 rpm	cw0–cw2, cw2–cw0, cw1–cw3, cw3–cw1
CW2	67 rpm	cw0–cw3, cw3–cw0

**Table 5:** Domain generalization tasks of the WT dataset.

Task Group	Rotational-Speed Difference	Domain Generalization Tasks
WT0	5 Hz	wt1–wt0, wt0–wt1, wt2–wt1, wt1–wt2, wt3–wt2, wt2–wt3, wt4–wt3, wt3–wt4, wt5–wt4, wt4–wt5, wt6–wt5, wt5–wt6, wt7–wt6, wt6–wt7
WT1	10 Hz	wt2–wt0, wt0–wt2, wt3–wt1, wt1–wt3, wt4–wt2, wt2–wt4, wt5–wt3, wt3–wt5, wt6–wt4, wt4–wt6, wt7–wt5, wt5–wt7
WT2	15 Hz	wt3–wt0, wt0–wt3, wt4–wt1, wt1–wt4, wt5–wt2, wt2–wt5, wt6–wt3, wt3–wt6, wt7–wt4, wt4–wt7
WT3	20 Hz	wt4–wt0, wt0–wt4, wt5–wt1, wt1–wt5, wt6–wt2, wt2–wt6, wt7–wt3, wt3–wt7
WT4	25 Hz	wt5–wt0, wt0–wt5, wt6–wt1, wt1–wt6, wt7–wt2, wt2–wt7
WT5	30 Hz	wt6–wt0, wt0–wt6, wt7–wt1, wt1–wt7
WT6	35 Hz	wt7–wt0, wt0–wt7

**Table 6:** Accuracy comparison on the CWRU bearing dataset.

Model	CW0	CW1	CW2	Task-Wise Mean	Overall Average
CNN	94.10	82.53	80.73	85.79	88.02
DANN	97.16	91.79	94.68	94.54	94.95
CIDA	97.32	89.70	91.79	92.94	93.86
Mixstyle	96.01	84.38	84.37	88.25	90.19
MSDGN	95.93	92.58	89.98	92.83	93.82
SDGN	**97.49**	**97.09**	86.48	93.69	95.52
WCIGDG (ours)	97.06	96.12	**97.04**	**96.74**	**96.74**

**Table 7:** Accuracy comparison on the WT planetary gearbox dataset.

Model	WT0	WT1	WT2	WT3	WT4	WT5	WT6	Task-Wise Mean	Overall Average
CNN	89.11	82.04	75.04	65.78	55.53	44.88	40.57	64.71	73.26
DANN	88.03	84.63	74.14	62.35	59.49	47.64	46.11	66.06	73.71
CIDA	90.22	83.50	75.78	63.01	53.42	38.52	36.07	62.93	72.75
Mixstyle	89.99	82.31	75.86	65.68	56.28	47.23	45.49	66.12	74.09
MSDGN	92.94	87.62	83.38	68.19	51.48	37.08	31.35	64.58	75.93
SDGN	**95.65**	89.25	79.68	**75.98**	57.01	43.58	45.08	69.46	78.95
WCIGDG (ours)	90.08	**89.31**	**82.75**	74.18	**63.80**	**55.33**	**68.85**	**74.90**	**80.28**

**Table 8:** F1-Score comparison on the CWRU bearing dataset.

Model	CW0	CW1	CW2	Task-Wise Mean	Overall Average
CNN	93.17	78.69	73.73	81.86	85.10
DANN	95.23	84.74	92.38	90.78	91.26
CIDA	95.39	81.65	86.58	87.88	89.34
Mixstyle	95.44	78.74	79.93	84.71	87.29
MSDGN	95.29	90.38	86.79	90.82	92.24
SDGN	**96.67**	**96.37**	79.59	90.88	93.72
WCIGDG (ours)	96.39	96.32	**96.86**	**96.52**	**96.44**

**Table 9:** F1-Score comparison on the WT planetary gearbox dataset.

Model	WT0	WT1	WT2	WT3	WT4	WT5	WT6	Task-Wise Mean	Overall Average
CNN	89.24	81.22	73.58	62.28	50.60	39.80	34.19	61.56	71.24
DANN	87.53	83.39	73.33	58.84	58.41	42.14	43.77	63.91	72.08
CIDA	89.90	82.09	74.35	59.96	50.15	34.13	30.87	60.21	70.82
Mixstyle	89.88	81.13	74.24	62.35	51.32	41.76	38.53	62.74	71.88
MSDGN	92.38	86.04	81.70	64.62	47.10	28.82	25.63	60.90	73.37
SDGN	**95.51**	88.86	78.72	**74.65**	54.05	39.54	39.38	67.24	77.66
WCIGDG (ours)	90.16	**89.13**	**81.84**	72.48	**61.68**	**54.32**	**69.78**	**74.20**	**79.59**

**Table 10:** Design of ablation experiments. A check mark (✓) indicates that the component is included, whereas an en dash (–) indicates that it is excluded.

Method	Domain Index Predictor	Adversarial Training of Domain Index Predictor	Multiple Domain Index Predictors
M1	–	–	–
M2	✓	–	–
M3	✓	✓	–
WCIGDG	✓	✓	✓

**Table 11:** Ablation results on the CWRU dataset.

Method	CW0	CW1	CW2	Task-Wise Mean	Overall Average
M1	91.78	78.36	77.16	82.43	84.87
M2	91.36	87.01	77.81	85.39	87.65
M3	**97.97**	90.89	93.64	94.17	94.89
WCIGDG	97.06	**96.12**	**97.04**	**96.74**	**96.74**

**Table 12:** Ablation results on the WT dataset.

Model	WT0	WT1	WT2	WT3	WT4	WT5	WT6	Task-Wise Mean	Overall Average
M1	**90.08**	83.03	74.55	65.32	54.03	41.50	38.32	63.83	73.08
M2	88.20	84.67	75.78	67.62	53.48	47.03	45.70	66.07	74.11
M3	89.75	82.24	77.38	65.73	59.29	52.87	48.16	67.92	75.12
WCIGDG	**90.08**	**89.31**	**82.75**	**74.18**	**63.80**	**55.33**	**68.85**	**74.90**	**80.28**

**Table 13:** Comparison of domain-index normalization schemes on the WT dataset.

Normalization Scheme	Accuracy on Task wt3–wt0 (%)
Min-Max (0–1)	95.08
Z-score Standardization	89.75
Logarithmic Transformation	79.10

**Table 14:** Relationship between rotational speed gap and distribution shift.

Task	wt0–wt1	wt0–wt2	wt0–wt3	wt0–wt4	wt0–wt5	wt0–wt6	wt0–wt7
Rotational-Speed Difference	5 Hz	10 Hz	15 Hz	20 Hz	25 Hz	30 Hz	35 Hz
Wasserstein Distance	0.35	0.63	0.99	1.44	1.82	2.26	2.55

**Table 15:** Average training duration per transfer task.

Model	Training Time (s)	
	CWRU	WT
CNN	84.80	87.10
DANN	90.03	441.81
CIDA	91.34	405.99
Mixstyle	95.99	93.31
MSDGN	341.89	448.44
SDGN	214.79	376.64
WCIGDG (ours)	188.04	395.55

**Table 16:** Robustness analysis of WCIGDG under noisy domain indices.

Noise Std. Dev. (Hz)	Perturbation Ratio (%)	Accuracy of Task wt3–wt0 (%)
0	0%	95.08
0.05	1%	95.90
0.25	5%	92.21
0.50	10%	90.08
1.00	20%	90.98
2.50	50%	90.57
5.00	100%	79.51

**Table 17:** Interpolation and extrapolation analysis on WT task wt2–wt6.

Setting	Available Indices for Predictor Training	Accuracy (%)	F1-Score
CNN baseline	No domain-index guidance	58.19	51.72
Interpolation	wt0–wt5, wt7	77.46	77.20
Extrapolation-1	wt0–wt5	71.72	71.07
Extrapolation-2	wt0–wt4	65.16	62.63
Extrapolation-3	wt0–wt3	64.75	61.28
Extrapolation-4	wt0–wt2	61.48	56.50

## Data Availability

The public datasets (CWRU, WT) analyzed in this study can be found using the following links: https://engineering.case.edu/bearingdatacenter/download-data-file (accessed on 25 January 2026); https://github.com/Liudd-BJUT/WT-planetary-gearbox-dataset (accessed on 25 January 2026).
